# TRAJECTORIES OF SUBJECTIVE MEMORY IMPAIRMENT AND OBJECTIVE COGNITIVE DECLINE IN U.S. OLDER ADULTS

**DOI:** 10.1093/geroni/igz038.743

**Published:** 2019-11-08

**Authors:** Hanzhang Xu, Matthew E Dupre, Bei Wu

**Affiliations:** 1 Duke University, Durham, North Carolina, United States; 2 Duke University School of Medicine, durham, North Carolina, United States; 3 new York University Rory Meyers College of Nursing, New York, New York, United States

## Abstract

We examined the dual trajectories of subjective memory impairment (SMI) and objective cognitive decline and their associated factors in U.S. older adults. We used data from the Health and Retirement Study which includes a nationally representative sample of 19,408 Americans age 65 and older from 1998 to 2016. Trajectories of SMI and objective cognitive decline were simultaneously characterized using a group-based trajectory model and multinomial logistic regressions were used to assess factors associated with the dual-trajectory typologies. Four dual-trajectories were identified: “minimal SMI and stable-low cognitive decline” (33.1% of respondents); “minimal SMI with accelerated cognitive decline” (28.2%); “significant SMI with moderate cognitive decline” (21.0%); and “moderate SMI with steady cognitive decline” (17.6%). Being male, minority, low educated, living alone, and having comorbidities were associated with trajectories featuring greater SMI or more rapid deterioration in cognition. The results suggest complex co-occurring changes in subjective memory and objective cognition in older adults.

